# Maintenance pharmacotherapy after electroconvulsive therapy in inpatients with major depressive disorder: 198 prescriptions in a real-world clinical setting

**DOI:** 10.1186/s12888-025-07445-4

**Published:** 2025-10-09

**Authors:** Shun Igarashi, Takashi Tsuboi, Naomi Hasegawa, Shinichiro Ochi, Kazutaka Ohi, Kentaro Fukumoto, Jun-ichi Iga, Hiroyuki Muraoka, Hitoshi Iida, Fumitoshi Kodaka, Shusuke Numata, Kayo Ichihashi, Hiroko Kashiwagi, Toshinori Nakamura, Hirotaka Yamagata, Masahiro Takeshima, Tatsuya Nagasawa, Junya Matsumoto, Hisashi Yamada, Hikaru Hori, Ken Inada, Norio Yasui-Furukori, Ryota Hashimoto, Koichiro Watanabe

**Affiliations:** 1 Department of Psychiatry, National Center Hospital, National Center of Neurology and Psychiatry, Kodaira, Japan; 2 Department of Neuropsychiatry, Kyorin University School of Medicine, 6-20-2 Shinkawa, Mitaka-shi, Tokyo, 181-8611 Japan; 3 Department of Pathology of Mental Diseases, National Institute of Mental Health, National Center of Neurology and Psychiatry, Kodaira, Japan; 4 Department of Neuropsychiatry, Molecules and Function, Ehime University Graduate School of Medicine, Matsuyama, Japan; 5 Department of Psychiatry, Gifu University Graduate School of Medicine, Gifu, Japan; 6 Department of Neuropsychiatry, School of Medicine, Iwate Medical University, Morioka, Japan; 7 Department of Psychiatry, Kitasato University School of Medicine, Sagamihara, Japan; 8 Department of Psychiatry, Faculty of Medicine, Fukuoka University, Fukuoka, Japan; 9 Department of Psychiatry, The Jikei University School of Medicine, Tokyo, Japan; 10 Department of Psychiatry, Graduate School of Biomedical Science, Tokushima University, Tokushima, Japan; 11 Department of Neuropsychiatry, University of Tokyo Hospital, Tokyo, Japan; 12 Department of Forensic Psychiatry, National Center of Neurology and Psychiatry, National Center Hospital, Kodaira, Japan; 13 Department of Psychiatry, Shinshu University School of Medicine, Matsumoto, Japan; 14 Department of Neuropsychiatry, Akita University Graduate School of Medicine, Akita, Japan; 15 Department of NeuroPsychiatry, Kanazawa Medical University, Kanazawa, Japan; 16 Department of Neuropsychiatry, Hyogo Medical University, Nishinomiya, Japan; 17 Department of Psychiatry, Dokkyo Medical University School of Medicine, Mibu, Japan

**Keywords:** Major depressive disorder, Electroconvulsive therapy, Maintenance pharmacotherapy, Antidepressants, Antipsychotics, Mood stabilizers.

## Abstract

**Background:**

Although antidepressant monotherapy is recommended for patients with major depressive disorder (MDD), they often do not respond to it, necessitating alternatives such as electroconvulsive therapy (ECT). However, maintenance pharmacotherapy after ECT has remained unestablished. This study, conducted at 240 facilities throughout Japan, aimed to explore maintenance pharmacotherapy after ECT for 3,749 inpatients with MDD.

**Methods:**

The patients were divided into two groups, one that underwent ECT (ECT group, *N* = 521) and another that did not (non-ECT group, *N* = 3,273), for the comparison of clinical characteristics and prescription details at discharge. The primary outcome of this study was the prescription rate of antidepressant monotherapy at discharge, while the secondary outcomes included prescription rates of specific combination regimens, such as antidepressant plus lithium.

**Results:**

We identified 198 prescription patterns involving antidepressants in the ECT group. Analysis by drug category revealed distinctive patterns: there was no statistically significant difference in prescription rates for antidepressant monotherapy between the ECT and non-ECT groups (*N* = 118, 22.6% vs. *N* = 932, 28.4%). In contrast, the prescription rate for the combination of antidepressant and antipsychotic medications was significantly higher in the ECT group (*N* = 188, 36.0% vs. *N* = 941, 28.7%). The combination of antidepressant and mood stabilizer was also more frequent in the ECT group (*N* = 35, 6.7% vs. *N* = 130, 3.9%), although this difference did not reach statistical significance after Bonferroni correction. At the drug level, additional distinctive patterns emerged: among antidepressant monotherapies, nortriptyline use was significantly more common in the ECT group (*N* = 9, 1.7% vs. *N* = 11, 0.3%). For mood stabilizers, restricting the analysis to lithium revealed a markedly higher rate in the ECT group (*N* = 30, 5.7% vs. *N* = 35, 1.0%).

**Conclusions:**

These findings highlight the complexity of treatment decisions managing of MDD after ECT and emphasize the need for structured prospective research on the effectiveness of specific maintenance pharmacotherapies after ECT.

**Supplementary Information:**

The online version contains supplementary material available at 10.1186/s12888-025-07445-4.

## Background

Although major depressive disorder (MDD) is a common disease, 30% of patients do not achieve remission with the continuation of standard pharmacotherapy [1]. Electroconvulsive therapy (ECT) is not only indicated for patients with severe depression with suicidal ideation, psychotic depression, and depression with catatonia but is also indicated for patients with treatment-resistant depression, with a reported response rate of 60% [2]. A 20% relapse rate after one year has been reported in patients with MDD who have achieved remission with pharmacotherapy rather than ECT [3]. However, even with continued treatment with antidepressants alone as maintenance pharmacotherapy after ECT, previous studies reported a 50% relapse rate after 12 months [4]. Therefore, the optimal maintenance pharmacotherapy after ECT should be considered.

Since patients with MDD who are indicated for or respond to ECT may have different backgrounds from those with depression who respond well to antidepressants, treatment with antidepressants alone may be insufficient for maintenance pharmacotherapy after ECT. Consistent with this rationale, a large population-based cohort showed that receipt of inpatient ECT is more likely among patients with psychotic depression and with greater depressive symptom burden, underscoring systematic differences in baseline clinical characteristics [5]. Furthermore, a meta-analysis demonstrated that psychotic features and older age predict higher odds of ECT response and remission, and that higher baseline severity predicts response [6]. These baseline differences are likely to influence the choice of maintenance pharmacotherapy; therefore, examining whether differences in post-discharge regimens between ECT and non-ECT patients reflect ECT per se or underlying clinical characteristics is clinically important. For example, guidelines recommend antidepressant–antipsychotic combinations for psychotic features and lithium augmentation for recurrent, severe, or treatment-resistant courses [7–9].

Several randomized controlled trials (RCTs) on maintenance pharmacotherapy after ECT have reported lower relapse rates with the combination therapy of lithium and nortriptyline or venlafaxine than with antidepressant monotherapy, suggesting that the combination of antidepressants and lithium may be useful as maintenance pharmacotherapy after ECT [10, 11]. However, owing to the various limitations of both RCTs: study duration, number of subjects, and lack of blindness, these results may not directly apply to maintenance pharmacotherapy after ECT in real-world practice.

In real-world settings, there is a lack of research focusing on maintenance pharmacotherapy after ECT in patients with MDD. In addition, most prior studies have focused solely on ECT-treated cohorts and lacked a non-ECT comparison group; nationwide real-world data directly comparing ECT and non-ECT patients with MDD remain scarce. A previous observational study examining the prescription of maintenance pharmacotherapy after ECT included 255 patients with MDD, bipolar disorder, schizophrenia, and schizoaffective disorder in Japan [12]. The study found that 109 patients (42%) used antidepressants as maintenance medications after ECT, 153 (60%) used mood stabilizers, and 176 (69%) used antipsychotics. Among the mood stabilizer users, lithium was used in 91 (35%) patients. The limitations of the study included the inclusion of various illnesses, the lack of investigation into the rate of concomitant use of antidepressants, and the fact that the study was conducted at a single center. Another previous multicenter study in the U.S. and Canada investigated psychotropic prescriptions at the end of acute ECT in 163 patients with depression and psychotic symptoms [13]. The study showed that the percentage of prescriptions at each center ranged from 3.6 to 23.8% for antidepressant monotherapy, 0 to 5.5% for the combination of antidepressants and lithium, and 43.6 to 63.2% for the combination of antidepressants and antipsychotics. The results indicated that the combination of antidepressants and antipsychotics was the most commonly used pharmacotherapy after ECT. However, the limitations of the study included the inclusion of patients with psychotic depression and the inter-institutional differences in the results, with the percentage of lithium prescriptions differing significantly from those in the Japanese study described above.

To address this gap, we used survey items collected through the nationwide EGUIDE project to directly compare maintenance pharmacotherapy regimens at discharge between ECT and non-ECT inpatients with MDD. We included a contemporaneous non-ECT inpatient comparison group to provide a real-world benchmark for post-discharge prescribing. This design helps contextualize whether observed differences are specific to ECT or reflect broader prescribing patterns among inpatients with MDD, while not implying causality.

In this study, we aimed to determine whether discharge maintenance pharmacotherapy regimens differ between inpatients with MDD who underwent ECT and those who did not. we hypothesized that the prescription rate of antidepressant monotherapy as maintenance pharmacotherapy after ECT would be low and that of combination therapy with antidepressants and mood stabilizers (e.g., lithium) or antipsychotics would be high. Therefore, we conducted a large-scale retrospective study to investigate the prescription patterns of maintenance pharmacotherapy after ECT in hospitalized patients with MDD.

## Methods

This study used data from 240 facilities participating in the EGUIDE (Effectiveness of Guidelines for Dissemination and Education in Psychiatric Treatment) project from 2016 to 2019. The project aims to evaluate whether various treatment modalities are concordant with clinical guideline recommendations. As part of this project, annual chart reviews are conducted at participating facilities, and psychiatrists review medical records and enter information into standardized forms. The details of the EGUIDE project have been published in several previous studies [14–16]. The inclusion criteria were those with a first admission during the study period and a diagnosis of MDD at discharge. The exclusion criteria were patients whose prescriptions were not entered immediately prior to admission or at the time of discharge and patients for whom the reliability of the data was determined to be uncertain.

In the project, psychiatrists at each participating site reviewed medical records and entered the survey items into standardized forms, which were then double-checked by data managers. Consequently, survey items on age, sex, and prescription at discharge were complete. Depressive severity (mild, moderate, severe) and the presence of psychotic features were intended to be extracted from the discharge diagnostic specifiers documented in the medical record, but these survey items were occasionally missing because some of the treating physicians did not record the specifiers in the discharge summary or medical records. ECT status (yes/no) was abstracted from the inpatient medical record and defined as ≥ 1 ECT session during the index hospitalization. All other eligible MDD inpatients with no ECT recorded comprised the non-ECT group. However, detailed procedural survey items on ECT (e.g., type of anesthetic agent, electrode placement, and the number of ECT sessions) were not collected in the chart review. Assignment was non-random and no matching was performed.

Patients who met the above criteria were divided into two groups, one that underwent ECT (ECT group) and another that did not undergo ECT (non-ECT group), to compare the clinical characteristics (age, sex, and depressive severity) and prescription details at discharge.

The prescription categories compared were twenty-three antidepressants, four mood stabilizers, thirty-five typical antipsychotics, and nine atypical antipsychotics approved in Japan. Anxiolytics, sleeping medications, and anticholinergics were not investigated in this study. Additionally, the prescription rates for each combination category were compared. Specifically, the following were examined at the time of discharge: antidepressant monotherapy; antidepressant polytherapy; combination therapy with antidepressants and mood stabilizers; combination therapy with antidepressants and atypical antipsychotics; combination therapy with antidepressants and typical antipsychotics; combination therapy with antidepressants, atypical antipsychotics, and typical antipsychotics; combination therapy with antidepressants, mood stabilizers, and atypical antipsychotics; combination therapy with antidepressants, mood stabilizers, and typical antipsychotics; combinations with antidepressants, mood stabilizers, and typical antipsychotics; and combinations with antidepressants, mood stabilizers, atypical antipsychotics, and typical antipsychotics. In this study, prescriptions at discharge were defined as maintenance pharmacotherapy after ECT. This definition assumes that discharge generally occurs when the patient’s condition has improved and that such prescriptions often remain unchanged for some time; however, whether these regimens were continued post-discharge or reflected a stable maintenance plan was not confirmed.

The primary outcome in this study was the between-group difference in the proportion of patients, at discharge, receiving each of three pre-specified categories: (i) antidepressant monotherapy, (ii) antidepressant and an atypical antipsychotic, and (iii) antidepressant and mood stabilizer. Secondary outcomes included other category combinations and selected drug-level regimens (e.g., nortriptyline monotherapy; venlafaxine–lithium; nortriptyline–lithium). In particular, we conducted additional analyses focusing on combinations involving lithium within the mood stabilizer category.

Statistical analyses were performed using IBM SPSS Statistics 28.0.0 (190) (IBM Japan, Tokyo, Japan). Student’s t-test and chi-square test were used for statistical analysis. Bonferroni’s correction was used to adjust for multiple comparisons in all chi-square tests. Statistical methods were used to compare the two groups when the ECT group exceeded 1% of the total number of patients, i.e., *N* = 6 or more.

This study was approved by the Ethics Committee of the National Center of Neurology and Psychiatry. The study was conducted in accordance with the Declaration of Helsinki.

## Results

A total of 3,749 patients (521 in the ECT group and 3,273 in the non-ECT group) participated in this study. The mean age was significantly higher in the ECT group (65.6 ± 12.8 years) than in the non-ECT group (56.8 ± 18.1 years) (*p* = < 0.0014). However, the sex (male ratio) was not significantly different between both groups (ECT group, 32.2%; non-ECT group, 35.4%). The prescription rate of antidepressants did not differ significantly between the ECT (85.0%) and non-ECT (83.8%) groups.

In the ECT group, the distribution of depressive severity was: mild 0.2% (1/521), moderate 13.1% (68/521), and severe 23.2% (121/521), with psychotic features present in 22.4% (117/521); severity/psychosis specifiers were missing in 41.1% (214/521). In the non-ECT group, the distribution was: mild 3.0% (98/3,273), moderate 24.1% (788/3,273), and severe 12.7% (416/3,273), with psychotic features present in 5.9% (192/3,273); missing in 54.4% (1,779/3,273).

A detailed prescription assessment revealed that the ECT group had a total of 198 prescription choices for antidepressant monotherapy and combination therapy, including antidepressants [see Additional file].

For the pre-specified primary comparisons, regarding the comparison between the two groups by category, there were no statistically significant differences in terms of antidepressant monotherapy, although there was a tendency for less prescription of this monotherapy in the ECT group (*N* = 118, 22.6%) compared to the non-ECT group (*N* = 932, 28.4%). Regarding the combination treatment with antidepressants and atypical antipsychotics, the ECT group (*N* = 188, 36.0%) was significantly higher than the non-ECT group (*N* = 941, 28.7%) (p = < 0.0014). Moreover, there were no significant differences between both groups in other category combinations (Table 1).

**Table 1:** Rate of prescriptions at discharge

	ECT group	Non-ECT group	*P*	Effect size	
	(*N* = 521)	(*N* = 3,273)		OR	95% CI
Use of AD	443 (85.0)	2,743 (83.8)	0.48	1.1	0.85–1.42
AD monotherapy	118 (22.6)	932 (28.4)	0.006	0.74	0.59–0.92
AD polytherapy	47 (9.0)	358 (10.9)	0.18	0.81	0.59–1.11
AD + MS	35 (6.7)	130 (3.9)	0.004	1.74	1.18–2.56
AD + AAP	188 (36.0)	941 (28.7)	< 0.0014*	1.4	1.15–1.70
AD + TAP	18 (3.4)	135 (4.1)	0.47	0.83	0.50–1.37
AD + AAP + TAP	2 (0.3)	56 (1.7)	-		
AD + MS + AAP	32 (6.1)	151 (4.6)	0.13	1.35	0.91–2.00
AD + MS + TAP	2 (0.3)	24 (0.7)	-		
AD + MS + AAP + TAP	1 (0.1)	16 (0.4)	-		
Non-use of AD	78 (14.9)	530 (16.1)			

Data show number of subjects (percentage)

Statistical methods were used to compare the two groups when the ECT group exceeded 1% of the total number of patients

*AD* Antidepressants, *AAP* Atypical antipsychotics, *CI* Confidence interval, *ECT* Electroconvulsive therapy, *MS* Mood stabilizer, *OR* Odds ratio, *TAP* Typical antipsychotics.

Chi-squared test were adjusted for multiple comparisons by Bonferroni‘s correction, and a p value < 0.0014 was defined as significant and marked with *. Odds ratios and 95% CIs were calculated for ECT group vs. non-ECT group

Secondary analyses included other category combinations and selected drug-level regimens.

A comparison of the content of each prescription in the antidepressant monotherapy and antidepressant polytherapy groups was presented (Table 2). Although nortriptyline monotherapy was significantly higher in the ECT group (*N* = 9, 1.7%) than in the non-ECT group (*N* = 11, 0.3%) (p = < 0.0014), there were no significant differences between the two groups for the other antidepressant monotherapies [see Table S1, Additional file]. Moreover, no significant differences were observed between the two groups for any combination of two or more antidepressants [see Table S2, Additional file].

**Table 2:** Prescriptions of AD at discharge

	ECT group	Non-ECT group	*P*	Effect size	
	(*N* = 521)	(*N* = 3,273)		OR	95% CI
AD (monotherapy)					
mirtazapine	37 (7.1)	334 (10.2)	0.008	0.67	0.47–0.96
escitalopram	12 (2.3)	147 (4.4)	0.021	0.5	0.28–0.91
duloxetine	11 (2.1)	103 (3.1)	0.19	0.66	0.35–1.24
venlafaxine	11 (2.1)	70 (2.2)	0.96	0.99	0.52–1.88
nortriptyline	9 (1.7)	11 (0.3)	< 0.0014*	5.21	2.15–12.64
paroxetine	8 (1.5)	60 (1.8)	0.63	0.83	0.38–1.80
sertraline	7 (1.3)	69 (2.1)	0.24	0.62	0.29–1.38
AD (polytherapy)					
duloxetine + mirtazapine	9 (1.7)	56 (1.7)	0.97	1.01	0.50–2.08
escitalopram + mirtazapine	9 (1.7)	34 (1.0)	0.16	1.63	0.77–3.23

Data show number of subjects (percentage)

In each medication, this table only shows prescriptions of 1% or more for ECT group. The supplement listed a prescription of 1% or less

*AD* Antidepressants, *CI* Confidence interval, *ECT* Electroconvulsive therapy, *OR* Odds ratio

Chi-squared test were adjusted for multiple comparisons by Bonferroni‘s correction, and a p value < 0.0014 was defined as significant and marked with *. Odds ratios and 95% CIs were calculated for ECT group vs. non-ECT group

A comparison between the two groups regarding 2 × 2 combinations of antidepressants and mood stabilizers (each monotherapy and polytherapy) was presented (Table 3). The combination of antidepressant monotherapy and mood stabilizer monotherapy was significantly higher in the ECT group (*N* = 33, 6.3%) than in the non-ECT group (*N* = 109, 3.3%) (p = < 0.0014). However, the other combinations were not significantly different between both groups. When examining the combination of antidepressant monotherapy and mood stabilizer monotherapy by each mood stabilizer, the combination of antidepressant monotherapy and lithium was significantly higher in the ECT group (*N* = 30, 5.7%) than in the non-ECT group (*N* = 35, 1.0%). Further categorization of antidepressants down to specific prescriptions, there was no significant difference between the ECT group and the non-ECT group except for the combination of venlafaxine and lithium (*N* = 7, 1.3% vs. *N* = 4, 0.1%), all of which were less than 1.3% in the ECT groups [see Table S3, Additional file]. There was no significant difference between the two groups regarding the combinations involving antidepressant monotherapy and other mood stabilizers.

**Table 3:** Prescriptions for combination therapy with AD + MS at discharge

	ECT group	Non-ECT group	*P*	Effect size	
	(*N* = 521)	(*N* = 3,273)		OR	95% CI
AD (monotherapy) + MS (monotherapy)	33 (5.7)	109 (3.3)	< 0.0014*	1.96	1.31–2.93
Lithium	30 (5.7)	35 (1.0)	< 0.0014*	3.44	3.44–9.29
Other MSs	3 (0.5)	74 (2.2)	-		
AD (polytherapy) + MS (monotherapy)	2 (0.3)	14 (0.5)	-		
AD (monotherapy) + MS (polytherapy)	0 (0.0)	5 (0.1)	-		
AD (polytherapy) + MS (polytherapy)	0 (0.0)	2 (0.0)	-		

Data show number of subjects (percentage)

In each medication, this table only shows prescriptions of 1% or more for ECT group. The supplement listed a prescription of 1% or less

*AD* Antidepressants, *CI* Confidence interval, *MS* Mood stabilizer, *ECT* Electroconvulsive therapy, *OR* Odds ratio

Statistical methods were used to compare the two groups when the ECT group exceeded 1% of the total number of patients Chi-squared test were adjusted for multiple comparisons by Bonferroni‘s correction, and a p value < 0.0014 was defined as significant and marked with *. Odds ratios and 95% CIs were calculated for ECT group vs. non-ECT group

A comparison between the two groups regarding the 2 × 2 combinations of antidepressants and atypical antipsychotics (each monotherapy and polytherapy) was presented (Table 4). The combination of antidepressant polytherapy and atypical antipsychotic monotherapy was significantly higher in the ECT group (*N* = 59, 11.3%) than in the non-ECT group (*N* = 232, 7.0%) (p = < 0.0014). However, other combinations were not significantly different between the two groups, although the combination of antidepressant monotherapy and atypical antipsychotic monotherapy was prescribed to 118 patients, exactly the same frequency as antidepressant monotherapy in the ECT group. Combinations involving antidepressant monotherapy and atypical antipsychotic monotherapy and combinations involving antidepressant polytherapy and atypical antipsychotic polytherapy were examined separately for each atypical antipsychotic, with no significant differences between the two groups for any atypical antipsychotic. When further divided into specific drug combinations, there were no significant differences between the ECT group and the non-ECT group, all of which were less than 2.3% in the ECT groups [see Table S4, Additional file].

**Table 4:** Prescriptions for combination therapy with AD and AAP at discharge

	ECT group	Non-ECT group	*P*	Effect size	
	(*N* = 521)	(*N* = 3,273)		OR	95% CI
AD (monotherapy) + AAP (monotherapy)	118 (22.6)	642 (19.6)	0.01	1.2	0.96–1.50
Quetiapine	41 (7.8)	221 (6.7)	0.35	1.18	0.83–1.67
Olanzapine	36 (6.9)	162 (4.9)	0.089	1.43	0.98–2.07
Aripiprazole	28 (5.3)	173 (5.2)	0.95	1.02	0.68–1.53
Risperidone	8 (1.5)	58 (1.7)	0.7	0.86	0.41–1.82
Other AAPs	5 (0.9)	28 (0.8)	-		
AD (monotherapy) + AAP (polytherapy)	11 (2.1)	46 (1.4)	0.21	1.51	0.78–2.94
AD (polytherapy) + AAP (monotherapy)	59 (11.3)	232 (7.0)	< 0.0014*	1.67	1.24–2.26
Aripiprazole	18 (3.4)	84 (2.5)	0.24	1.36	0.81–2.28
Quetiapine	18 (3.4)	72 (2.1)	0.08	1.59	0.94–2.69
Olanzapine	13 (2.4)	47 (1.4)	0.072	1.76	0.94–3.27
Risperidone	5 (0.9)	18 (0.5)	-		
Other AAPs	5 (0.9)	11 (0.3)	-		
AD (polytherapy) + AAP (polytherapy)	0 (0.0)	21 (0.6)	-		

Data show number of subjects (percentage)

In each medication, this table only shows prescriptions of 1% or more for ECT group (with the exception of risperidone). The supplement listed a prescription of 1% or less

No specific combination of AD (monotherapy) and AAP (polytherapy) showed more than 1% for ECT group

Statistical methods were used to compare the two groups when the ECT group exceeded 1% of the total number of patients

*AD* Antidepressants, *AAP* Atypical antipsychotics, *CI* Confidence interval, *ECT* Electroconvulsive therapy, OR Odds ratio

Chi-squared test were adjusted for multiple comparisons by Bonferroni‘s correction, and a p value < 0.0014 was defined as significant and marked with *. Odds ratios and 95% CIs were calculated for ECT group vs. non-ECT group

No combinations in the categories of antidepressants and typical antipsychotics; antidepressants, atypical antipsychotics, and typical antipsychotics; antidepressants, mood stabilizers, and atypical antipsychotics; antidepressants, mood stabilizers, and typical antipsychotics; and antidepressants, mood stabilizers, atypical antipsychotics, and typical antipsychotics were statistically examined because none of these combinations exceeded 1% in the ECT group [see Table S5-9, Additional file].

## Discussion

To the best of our knowledge, this is the first multicenter observational study on maintenance pharmacotherapy after ECT in inpatients with MDD in Japan. This study described the prescriptions in detail, which revealed 198 maintenance pharmacotherapy regimens after ECT, including antidepressants as monotherapy and psychotropic drugs in combination with antidepressants. In a categorical comparison, the ECT group showed a lower rate of antidepressant monotherapy prescriptions compared with the non-ECT group; however, this difference was not statistically significant. In contrast, there was a significantly higher rate of prescriptions for combination therapy with antidepressants and atypical antipsychotics in the ECT group. Further classification of monotherapy and polytherapy within each category showed that the combinations involving antidepressant polytherapy and atypical antipsychotic monotherapy and the combinations involving antidepressant monotherapy and mood stabilizer monotherapy were frequently used in the comparison between the two groups.

In this study, a large variety of nearly 200 prescription patterns was selected as maintenance pharmacotherapy for 521 patients who underwent ECT. The following factors may be associated with the variety in prescription patterns. First, there is no consistent consensus on maintenance pharmacotherapy after ECT, which is why national guidelines differ in their recommendations. According to the Japanese guidelines for the treatment of MDD, regarding maintenance pharmacotherapy after ECT, patients who achieve response or remission promptly with ECT can be maintained on novel antidepressants alone, whereas those with two or more recurrent episodes or severe depressive episodes are mildly recommended to use lithium in combination with antidepressants [7]. Canadian guidelines for the treatment of MDD strongly recommend the combination of antidepressants and lithium as maintenance pharmacotherapy after ECT [8], whereas the VA/DoD Clinical Practice Guidelines for its treatment suggest that treatment with antidepressants should be considered first after acute ECT and that continuous and maintenance ECT should be considered in cases where it is not well tolerated [9]. Thus, inconsistent guideline statements may have annoyed clinicians and influenced their prescribing patterns. Second, because depression is a highly heterogeneous disorder [17], its treatments may also be diverse. A survey conducted in the U.S. reported that antidepressants were not the treatment of choice, even for patients diagnosed with MDD [18]. In our previous EGUIDE project study, 53% of patients with MDD were treated with antipsychotics and 18% with mood stabilizers [19]. The greater variety of prescriptions observed in patients with MDD who underwent ECT may reflect the higher likelihood of severe or treatment-resistant conditions.

In one categorical comparison, the combination involving antidepressants and atypical antipsychotics was significantly more frequently used in the ECT group than in the non-ECT group. More specifically, the combination involving antidepressant polytherapy and atypical antipsychotic monotherapy was significantly different between the two groups, although it should be noted that this combination was prescribed to fewer patients than the combination of antidepressant monotherapy and atypical antipsychotic monotherapy in the ECT group. Alexopoulos et al. reported that 82% of mood disorder specialists listed a combination of antidepressants and antipsychotics as their first choice for maintenance pharmacotherapy after ECT in older patients with psychotic depression [20]. Since psychotic depression is the primary indication for ECT, a combination of antidepressants and antipsychotics is recommended as pharmacotherapy for patients who have or have not undergone ECT [21]. In our previous study, MDD severity was measured in only half of the patients, of whom only 14.1% had psychotic depression [22]. However, the results of that study may reflect patients with psychotic depression.

In another categorical comparison, the ECT group showed a higher prescription rate for combination therapy with antidepressants and mood stabilizers. Following the classification into monotherapy and polytherapy regarding these two drugs, the combination of antidepressant monotherapy and mood stabilizer monotherapy was used significantly more frequently in the ECT group than in the non-ECT group. The combination of antidepressant monotherapy and lithium monotherapy accounted for more than 90% of the prescriptions. Several previous studies have revealed the efficacy of lithium in combination with antidepressants, particularly nortriptyline and venlafaxine, as maintenance pharmacotherapy after ECT [10, 11]. Moreover, a meta-analysis that included these studies suggested that the lithium-use group was superior to the non-use group in reducing the risk of recurrence after acute ECT in patients with MDD [23], supporting the results of this study. In contrast, in this study, the combination of antidepressant monotherapy and lithium monotherapy as maintenance pharmacotherapy after ECT was limited to 5.7% of the patients in the ECT group. In Japan, lithium is used off-label for MDD, and if lithium were indicated for MDD, we might prescribe it more frequently. There is a lack of previous research on which types of patients with MDD are appropriate for pharmacotherapy as maintenance medications after ECT, and further studies are needed.

One of the strengths of this observational study is that it is the largest investigation of maintenance pharmacotherapy after ECT in a multicenter involving over 3,000 inpatients with MDD at 240 facilities nationwide, including more than 500 ECT cases. Another strength of this study is that because it was a real-world observational study, the results can be directly reflected in actual clinical practice, unlike intervention studies with many exclusion criteria. Moreover, because a control group was established, the influence of having undergone ECT or not on the subsequent choice of maintenance prescription could be considered.

However, this study had several limitations. First, as this study was conducted with a retrospective observational design, it was not possible to discuss causal relationships, and all findings should be interpreted as associations rather than evidence of causation. Second, prescriptions at discharge were defined as maintenance pharmacotherapy after ECT. However, this definition would not be problematic because discharge from the hospital is often taken as an indicator of an improved condition, and the prescription at discharge is generally unchanged for some time. Nevertheless, post-discharge continuation or regimen stability was not confirmed. Third, the extent to which depression with catatonic features and treatment-resistant depression (which are considered indications for ECT) were included in this study was not investigated. In addition, survey items for severity and psychotic features were missing for a substantial proportion of patients, particularly in the non-ECT group. The high rate of missing survey items may have introduced bias into the baseline comparisons and limited the ability to adjust for these survey items in the analyses. As such, these characteristics were summarized descriptively, and any observed differences between groups should be interpreted with caution. Fourth, psychotropic medications other than antidepressants, antipsychotics, and mood stabilizers were not examined. Fifth, MDD severity was not measured using standardized assessment scales. Sixth, survey items on prior depressive episodes, detailed procedural information on ECT, and the number of ECT sessions administered during the index hospitalization were not recorded, which precluded analyses considering treatment course or cumulative stimulus dose. In addition, the potential for selection bias is high, and any between-group differences should be interpreted as non-causal. Seventh, this study was conducted exclusively in Japan, which may limit the generalizability of the findings to other countries with different health care systems, ECT practices, and prescribing cultures.

## Conclusions

In conclusion, this study investigated the prescription patterns of maintenance pharmacotherapy after ECT in inpatients with MDD. It revealed that a wide variety of antidepressant therapies and their combinations with other medication categories were used. Given the variety of prescribing patterns, further structured prospective studies are warranted to evaluate the effectiveness of specific prescriptions for maintenance pharmacotherapy after ECT in patients with MDD.

## Supplementary Information

Supplementary Material 1: Table S1 Prescriptions of AD monotherapy at discharge, Table S2 Prescriptions of AD polytherapy at discharge, Table S3 Prescriptions for combination therapy with AD + MS at discharge, Table S4 Prescriptions for combination therapy with AD + AAP at discharge, Table S5 Prescriptions for combination therapy with AD + TAP at discharge, Table S6 Prescriptions for combination therapy with AD +AAP +TAP at discharge, Table S7 Prescriptions for combination therapy with AD +MS +AAP at discharge, Table S8 Prescriptions for combination therapy with AD +MS +TAP at discharge, and Table S9 Prescriptions for combination therapy with AD +MS +AAP + TAP at discharge Description of data: A detailed prescription assessment revealed that the ECT group had a total of 198 prescription choices for antidepressant monotherapy and combination therapy, including antidepressants.

## Data Availability

No datasets were generated or analysed during the current study.

## Abbreviations

AAP: atypical antipsychotics
AD: antidepressants
ECT: Electroconvulsive therapy
EGUIDE: Effectiveness of Guidelines for Dissemination and Education in Psychiatric Treatment
MDD: major depressive disorder
MS: mood stabilizer
RCTs: randomized controlled trials
TAP: typical antipsychotics
