# Supplementary material for: Maintenance pharmacotherapy after electroconvulsive therapy in inpatients with major depressive disorder: 198 prescriptions in a real-world clinical setting
Source: BMC Psychiatry. 2025 Oct 9;25:957. doi: 10.1186/s12888-025-07445-4 (PMC12512634; doi:10.1186/s12888-025-07445-4)
Supplement: Supplementary file 1 — Supplementary Material 1: Table S1 Prescriptions of AD monotherapy at discharge, Table S2 Prescriptions of AD polytherapy at discharge, Table S3 Prescriptions for combination therapy with AD + MS at discharge, Table S4 Prescriptions for combination therapy with AD + AAP at discharge, Table S5 Prescriptions for combination therapy with AD + TAP at discharge, Table S6 Prescriptions for combination therapy with AD +AAP +TAP at discharge, Table S7 Prescriptions for combination therapy with AD +MS +AAP at discharge, Table S8 Prescriptions for combination therapy with AD +MS +TAP at discharge, and Table S9 Prescriptions for combination therapy with AD +MS +AAP + TAP at discharge Description of data: A detailed prescription assessment revealed that the ECT group had a total of 198 prescription choices for antidepressant monotherapy and combination therapy, including antidepressants. [file 12888_2025_7445_MOESM1_ESM.pdf]

Table S1 Prescriptions of AD monotherapy at discharge

	ECT group (N=521)	Non-ECT group (N=3,273)
mirtazapine	37 (7.1)	334 (10.2)
escitalopram	12 (2.3)	147 (4.4)
duloxetine	11 (2.1)	103 (3.1)
venlafaxine	11 (2.1)	70 (2.2)
nortriptyline	9 (1.7)	11 (0.3)
paroxetine	8 (1.5)	60 (1.8)
sertraline	7 (1.3)	69 (2.1)
amitriptyline	4 (0.7)	11 (0.3)
amoxapine	4 (0.7)	10 (0.3)
trazodone	4 (0.7)	23 (0.7)
clomipramine	3 (0.5)	12 (0.3)
mianserin	3 (0.5)	8 (0.2)
milnacipran	2 (0.3)	9 (0.2)
paroxetine	2 (0.3)	33 (1.0)
fluvoxamine	1 (0.1)	17 (0.5)

Data show number of subjects (percentage).

The abbreviations are AD for antidepressants, and ECT for electroconvulsive therapy.

Table S2 Prescriptions of AD polytherapy at discharge

	ECT group (N=521)	Non-ECT group (N=3,273)
duloxetine + mirtazapine	9 (1.7)	56 (1.7)
escitalopram + mirtazapine	9 (1.7)	34 (1.0)
duloxetine + trazodone	3 (0.5)	28 (0.8)
amoxapine + trazodone	2 (0.3)	2 (0.0)
escitalopram + trazodone	2 (0.3)	17 (0.5)
nortriptyline + trazodone	2 (0.3)	0 (0.0)
mirtazapine + sertraline	2 (0.3)	30 (0.9)
trazodone + venlafaxine	2 (0.3)	11 (0.3)
mirtazapine + paroxetine + trazodone	1 (0.1)	1 (0.0)
mirtazapine + trazodone + venlafaxine	1 (0.1)	2 (0.0)
clomipramine + mirtazapine + sertraline	1 (0.1)	0 (0.0)
amoxapine + duloxetine	1 (0.1)	1 (0.0)
amoxapine + mirtazapine	1 (0.1)	2 (0.0)
clomipramine + venlafaxine	1 (0.1)	0 (0.0)
clomipramine + mianserin	1 (0.1)	1 (0.0)
duloxetine + sertraline	1 (0.1)	2 (0.0)
nortriptyline + paroxetine	1 (0.1)	1 (0.0)
nortriptyline + sertraline	1 (0.1)	0 (0.0)
mianserine + paroxetine	1 (0.1)	0 (0.0)
mirtazapine + paroxetine	1 (0.1)	6 (0.1)
mirtazapine + trazodone	1 (0.1)	0 (0.0)
mirtazapine+ venlafaxine	1 (0.1)	21 (0.6)
paroxetine + trazodone	1 (0.1)	12 (0.3)
sertraline +trazodone	1 (0.1)	13 (0.3)

Data show number of subjects (percentage).

The abbreviations are AD for antidepressants, and ECT for electroconvulsive therapy.

Table S3 Prescriptions for combination therapy with AD + MS at discharge

	ECT group (N=521)	Non-ECT group (N=3,273)
**AD (monotherapy) + lithium**		
venlafaxine	7 (1.3)	4 (0.1)
escitalopram	4 (0.7)	6 (0.1)
mirtazapine	4 (0.7)	10 (0.3)
paroxetine	4 (0.7)	0 (0.0)
duloxetine	2 (0.3)	3 (0.0)
sertraline	2 (0.3)	4 (0.1)
trazodone	2 (0.3)	3 (0.0)
mianserin	2 (0.3)	0 (0.0)
nortriptyline	2 (0.3)	2 (0.0)
fluvoxamine	1 (0.1)	1 (0.0)
**AD (monotherapy) + others**		
valproate + escitalopram	1 (0.1)	9 (0.2)
lamotrigine + mirtazapine	1 (0.1)	1 (0.0)
carbamazepine + escitalopram	1 (0.1)	2 (0.0)
**AD (polytherapy) + MS (monotherapy)**		
amoxapine + venlafaxine +lithium	2 (0.3)	0 (0.0)

Data show number of subjects (percentage).

The abbreviations are AD for antidepressants, ECT for electroconvulsive therapy, and MS for mood stabilizer.

Table S4 Prescriptions for combination therapy with AD + AAP at discharge

	ECT group (N=521)	Non-ECT group (N=3,273)
**AD (monotherapy) + AAP (monotherapy)**		
AD (monotherapy) + quetiapine		
mirtazapine	12 (2.3)	71 (2.1)
duloxetine	7 (1.3)	24 (0.7)
escitalopram	4 (0.7)	38 (1.1)
sertraline	4 (0.7)	27 (0.8)
venlafaxine	4 (0.7)	18 (0.5)
paroxetine	4 (0.7)	13 (0.3)
amoxapine	2 (0.3)	6 (0.1)
trazodone	1 (0.1)	9 (0.2)
clomipramine	1 (0.1)	3 (0.0)
nortriptyline	1 (0.1)	3 (0.0)
imipramine	1 (0.1)	0 (0.0)
AD (monotherapy) + olanzapine		
mirtazapine	10 (1.9)	49 (1.4)
duloxetine	5 (0.9)	27 (0.8)
paroxetine	5 (0.9)	13 (0.3)
sertraline	4 (0.7)	10 (0.3)
clomipramine	3 (0.5)	2 (0.0)
amitriptyline	3 (0.5)	1 (0.0)
escitalopram	2 (0.3)	35 (1.0)
trazodone	2 (0.3)	3 (0.0)
venlafaxine	1 (0.1)	12 (0.3)
amoxapine	1 (0.1)	2 (0.0)
AD (monotherapy) + aripiprazole		
mirtazapine	11 (2.1)	55 (1.6)
sertraline	4 (0.7)	19 (0.5)
escitalopram	3 (0.5)	28 (0.8)
paroxetine	3 (0.5)	10 (0.3)
duloxetine	2 (0.3)	22 (0.6)

venlafaxine	2 (0.3)	17 (0.5)
fluvoxamine	1 (0.1)	5 (0.1)
nortriptyline	1 (0.1)	2 (0.0)
milnacipran	1 (0.1)	1 (0.0)
AD (monotherapy) + risperidone		
mirtazapine	3 (0.5)	12 (0.3)
escitalopram	1 (0.1)	13 (0.3)
sertraline	1 (0.1)	4 (0.1)
venlafaxine	1 (0.1)	4 (0.1)
nortriptyline	1 (0.1)	1 (0.0)
clomipramine	1 (0.1)	0 (0.0)
AD (monotherapy) + asenapine		
mirtazapine	1 (0.1)	4 (0.1)
amitriptyline	1 (0.1)	0 (0.0)
AD (monotherapy) + perospirone		
trazodone	1 (0.1)	0 (0.0)
AD (monotherapy) + paliperidone		
escitalopram	1 (0.1)	1 (0.0)
AD (monotherapy) + brexpiprazole		
mirtazapine	1 (0.1)	1 (0.0)
**AD (monotherapy) + AAP (polytherapy)**		
escitalopram + olanzapine +quetiapine	2 (0.3)	2 (0.0)
duloxetine +olanzapine +quetiapine	2 (0.3)	0 (0.0)
escitalopram +aripiprazole +olanzapine	1 (0.1)	1 (0.0)
duloxetine +aripiprazole +olanzapine	1 (0.1)	0 (0.0)
duloxetine +aripiprazole +quetiapine	1 (0.1)	2 (0.0)
duloxetine +quetiapine + risperidone	1 (0.1)	0 (0.0)
mirtazapine +quetiapine + risperidone	1 (0.1)	1 (0.0)
mirtazapine +aripiprazole +olanzapine	1 (0.1)	0 (0.0)
venlafaxine +olanzapine +quetiapine	1 (0.1)	0 (0.0)
**AD (polytherapy) + AAP (monotherapy)**		
AD (polytherapy) + quetiapine		
mirtazapine + trazodone	2 (0.3)	7 (0.2)

mirtazapine + duloxetine	2 (0.3)	6 (0.1)
escitalopram + sertraline	1 (0.1)	0 (0.0)
duloxetine + escitalopram	1 (0.1)	2 (0.0)
duloxetine + trazodone	1 (0.1)	2 (0.0)
escitalopram + milnacipran	1 (0.1)	0 (0.0)
escitalopram + mirtazapine	1 (0.1)	6 (0.1)
amoxapine + mirtazapine	1 (0.1)	3 (0.0)
mirtazapine + mianserin	1 (0.1)	0 (0.0)
mirtazapine + nortriptyline	1 (0.1)	1 (0.0)
mirtazapine + paroxetine	1 (0.1)	1 (0.0)
mirtazapine + Venlafaxine	1 (0.1)	7 (0.2)
nortriptyline + paroxetine	1 (0.1)	0 (0.0)
paroxetine + trazodone	1 (0.1)	0 (0.0)
sertraline + amoxapine	1 (0.1)	0 (0.0)
sertraline + trazodone	1 (0.1)	1 (0.0)
AD (polytherapy) + olanzapine		
mirtazapine + trazodone	2 (0.3)	3 (0.0)
duloxetine + mirtazapine	2 (0.3)	8 (0.2)
amitriptyline + trazodone	1 (0.1)	0 (0.0)
amoxapine + fluvoxamine	1 (0.1)	0 (0.0)
amoxapine + mirtazapine	1 (0.1)	0 (0.0)
duloxetine + trazodone	1 (0.1)	0 (0.0)
duloxetine + mianserin	1 (0.1)	2 (0.0)
mirtazapine +duloxetine	1 (0.1)	0 (0.0)
mirtazapine + sertraline	1 (0.1)	1 (0.0)
mirtazapine + venlafaxine	1 (0.1)	6 (0.1)
sertraline + venlafaxine	1 (0.1)	0 (0.0)
AD (polytherapy) + aripiprazole		
duloxetine + mirtazapine	9 (1.7)	13 (0.3)
mirtazapine + clomipramine	2 (0.3)	0 (0.0)
duloxetine +escitalopram	1 (0.1)	2 (0.0)
escitalopram + trazodone	1 (0.1)	8 (0.2)
mirtazapine + amitriptyline	1 (0.1)	0 (0.0)
mirtazapine + sertraline	1 (0.1)	8 (0.2)
mirtazapine +trazodone	1 (0.1)	5 (0.1)

sertraline + venlafaxine	1 (0.1)	2 (0.0)
paroxetine + mianserin	1 (0.1)	0 (0.0)
AD (polytherapy) + risperidone		
amoxapine + trazodone	1 (0.1)	0 (0.0)
duloxetine + trazodone	1 (0.1)	1 (0.0)
mirtazapine + sertraline	1 (0.1)	1 (0.0)
mirtazapine + trazodone	1 (0.1)	2 (0.0)
paroxetine + trazodone	1 (0.1)	0 (0.0)
AD (polytherapy) + paliperidone		
amitriptyline +trazodone	1 (0.1)	0 (0.0)
AD (polytherapy) + perospirone		
duloxetine + mirtazapine + trazodone	1 (0.1)	1 (0.0)
mirtazapine +nortriptyline + sertraline	1 (0.1)	0 (0.0)
nortriptyline + trazodone	1 (0.1)	0 (0.0)
mirtazapine + trazodone	1 (0.1)	0 (0.0)

Data show number of subjects (percentage).

The abbreviations are AD for antidepressants, AAP for atypical antipsychotics, and ECT for electroconvulsive therapy.

Table S5 Prescriptions for combination therapy with AD + TAP at discharge

	ECT group (N=521)	Non-ECT group (N=3,273)
sulpiride ＋ duloxetine + mirtazapine	2 (0.3)	1 (0.0)
chlorpromazine + imipramine + mirtazapine	1 (0.1)	0 (0.0)
Levomepromazine ＋ trazodone＋ venlafaxine	1 (0.1)	2 (0.0)
sulpiride ＋ amoxapine ＋ duloxetine	1 (0.1)	0 (0.0)
chlorpromazine + mianserin + trazodone	1 (0.1)	0 (0.0)
chlorpromazine ＋ duloxetine + mirtazapine	1 (0.1)	0 (0.0)
sulpiride + paroxetine	1 (0.1)	2 (0.0)
sulpiride ＋ nortriptyline	1 (0.1)	0 (0.0)
sulpiride ＋ trazodone	1 (0.1)	4 (0.1)
sulpiride ＋ venlafaxine	1 (0.1)	2 (0.0)
sulpiride ＋ clomipramine	1 (0.1)	0 (0.0)
sulpiride ＋ mirtazapine	1 (0.1)	10 (0.3)
sulpiride ＋ sertraline	1 (0.1)	3 (0.0)
zotepine ＋ amitriptyline	1 (0.1)	0 (0.0)
levomepromazine ＋ duloxetine	1 (0.1)	3 (0.0)
tiapride ＋ mirtazapine	1 (0.1)	1 (0.0)
levomepromazine ＋ mirtazapine	1 (0.1)	5 (0.1)

Data show number of subjects (percentage).

The abbreviations are AD for antidepressants, ECT for electroconvulsive therapy, and TAP for typical antipsychotics.

Table S6 Prescriptions for combination therapy with AD +AAP +TAP at discharge

	ECT group (N=521)	Non-ECT group (N=3,273)
mirtazapine ＋ quetiapine + levomepromazine	1 (0.1)	0 (0.0)
mirtazapine ＋ olanzapine ＋ levomepromazine	1 (0.1)	2 (0.0)

Data show number of subjects (percentage).

The abbreviations are AD for antidepressants, AAP for atypical antipsychotics, ECT for electroconvulsive therapy, and TAP for typical antipsychotics.

Table S7 Prescriptions for combination therapy with AD +MS +AAP at discharge

	ECT group (N=521)	Non-ECT group (N=3,273)
quetiapine + mirtazapine + lithium	3 (0.5)	2 (0.0)
quetiapine + venlafaxine + lithium	2 (0.3)	1 (0.0)
olanzapine + mirtazapine + lithium	2 (0.3)	5 (0.1)
olanzapine + paroxetine + lithium	2 (0.3)	1 (0.0)
olanzapine + duloxetine + lithium	1 (0.1)	0 (0.0)
quetiapine ＋ risperidone ＋ mirtazapine + valproate	1 (0.1)	1 (0.0)
quetiapine ＋ risperidone ＋ mirtazapine + valproate	1 (0.1)	1 (0.0)
aripiprazole ＋ quetiapine ＋ duloxetine + lamotrigine	1 (0.1)	0 (0.0)
aripiprazole + duloxetine + mirtazapine + trazodone + lithium	1 (0.1)	0 (0.0)
aripiprazole + escitalopram + paroxetine + trazodone + valproate	1 (0.1)	0 (0.0)
aripiprazole + mianserin + milnacipran + valproate	1 (0.1)	0 (0.0)
aripiprazole + duloxetine + mirtazapine + lamotrigine	1 (0.1)	2 (0.0)
aripiprazole + escitalopram + lithium	1 (0.1)	1 (0.0)
aripiprazole + clomipramine + lithium	1 (0.1)	0 (0.0)
aripiprazole + mirtazapine + lamotrigine	1 (0.1)	0 (0.0)
olanzapine + mirtazapine + venlafaxine + lithium	1 (0.1)	1 (0.0)
olanzapine + milnacipran + lamotrigine	1 (0.1)	0 (0.0)
olanzapine + mirtazapine + lamotrigine	1 (0.1)	0 (0.0)
olanzapine + trazodone+ lamotrigine	1 (0.1)	1 (0.0)
quetiapine + amitriptyline + mirtazapine + nortriptyline + Lithium + valproate	1 (0.1)	0 (0.0)
quetiapine + mirtazapine + paroxetine + lithium	1 (0.1)	0 (0.0)
quetiapine + sertraline + lithium	1 (0.1)	2 (0.0)
quetiapine + amoxapine + lithium	1 (0.1)	1 (0.0)
quetiapine + fluvoxamine + lithium	1 (0.1)	0 (0.0)
quetiapine + nortriptyline +lithium	1 (0.1)	0 (0.0)
quetiapine + mirtazapine + lamotrigine	1 (0.1)	3 (0.0)
Risperidone + duloxetine + lithium	1 (0.1)	0 (0.0)

Data show number of subjects (percentage).

The abbreviations are AD for antidepressants, AAP for atypical antipsychotics, ECT for electroconvulsive therapy, and MS for mood stabilizer.

Table S8 Prescriptions for combination therapy with AD +MS +TAP at discharge

	ECT group (N=521)	Non-ECT group (N=3,273)
levomepromazine + venlafaxine + lithium	1 (0.1)	1 (0.0)
levomepromazine + paroxetine + Lithium	1 (0.1)	0 (0.0)

Data show number of subjects (percentage).

The abbreviations are AD for antidepressants, ECT for electroconvulsive therapy, MS for mood stabilizer, and TAP for typical antipsychotics.

Table S9 Prescriptions for combination therapy with AD +MS +AAP + TAP at discharge

	**ECT group (N=521)**	**Non-ECT group (N=3,273)**
sertraline + lithium +olanzapine ＋ levomepromazine	1 (0.1)	0 (0.0)

Data show number of subjects (percentage).

The abbreviations are AD for antidepressants, AAP for atypical antipsychotics, ECT for electroconvulsive therapy, MS for mood stabilizer, and TAP for typical antipsychotics.
